# Virulence genes, resistome and mobilome of Streptococcus suis strains isolated in France

**DOI:** 10.1099/mgen.0.001224

**Published:** 2024-03-28

**Authors:** Manon Dechêne-Tempier, Claire de Boisséson, Pierrick Lucas, Stéphanie Bougeard, Virginie Libante, Corinne Marois-Créhan, Sophie Payot

**Affiliations:** 1 Anses Laboratoire de Ploufragan-Plouzané-Niort, Unité Mycoplasmologie, Bactériologie et Antibiorésistance, BP53 22440 Ploufragan, France; 2 Université de Lorraine, INRAE, DynAMic, F-54000 Nancy, France; 3 Anses Laboratoire de Ploufragan-Plouzané-Niort, Unité Génétique Virale et Biosécurité, BP53 22440 Ploufragan, France; 4 Anses Laboratoire de Ploufragan-Plouzané-Niort, Unité Épidémiologie, santé et bien-être, BP53 22440 Ploufragan, France

**Keywords:** competence, integrative conjugative elements, mobilizable elements, resistance genes, *Streptococcus suis*, virulence

## Abstract

*Streptococcus suis* is a leading cause of infection in pigs, causing extensive economic losses. In addition, it can also infect wild fauna, and can be responsible for severe infections in humans. Increasing antimicrobial resistance (AMR) has been described in *S. suis* worldwide and most of the AMR genes are carried by mobile genetic elements (MGEs). This contributes to their dissemination by horizontal gene transfer. A collection of 102 strains isolated from humans, pigs and wild boars in France was subjected to whole genome sequencing in order to: (i) study their genetic diversity, (ii) evaluate their content in virulence-associated genes, (iii) decipher the mechanisms responsible for their AMR and their association with MGEs, and (iv) study their ability to acquire extracellular DNA by natural transformation. Analysis by hierarchical clustering on principal components identified a few virulence-associated factors that distinguish invasive CC1 strains from the other strains. A plethora of AMR genes (*n*=217) was found in the genomes. Apart from the frequently reported *erm*(B) and *tet*(O) genes, more recently described AMR genes were identified [*vga*(F)/*sprA*, *vat*(D)]. Modifications in PBPs/MraY and GyrA/ParC were detected in the penicillin- and fluoroquinolone-resistant isolates respectively. New AMR gene–MGE associations were detected. The majority of the strains have the full set of genes required for competence, i.e for the acquisition of extracellular DNA (that could carry AMR genes) by natural transformation. Hence the risk of dissemination of these AMR genes should not be neglected.

**Impact Statement**

The zoonotic pathogen *Streptococcus suis* is the causative agent of systemic diseases in pigs and humans, and thus poses economic and public health concerns. Increasing antimicrobial resistance (AMR) and carriage of the AMR determinants by mobile genetic elements (MGEs) have been reported for this bacterial species in many countries but data are scarce for strains isolated in France. Using whole genome sequencing, we analysed 102 strains of different serotypes isolated in France (91, eight and three strains isolated from pigs, humans and wild boars respectively). We found that strains isolated from the upper or lower respiratory tract can carry up to 64 virulence-associated genes. A total of 217 AMR genes were identified, most of them being carried by MGEs, in particular integrative mobilizable elements and integrative conjugative elements. New AMR gene–MGE associations were identified. Most of the strains have a full set of competence genes thus being theoretically able to take up AMR and virulence genes from other bacteria in the environment and serve as reservoir of genes for other species having this trait. Given the risk of dissemination of AMR to other bacterial species, this study highlights the need for reinforcing surveillance of AMR in *S. suis* not only for invasive strains but also for carriage strains.

## Data Summary

The authors confirm all supporting data, code and protocols have been provided within the article or through supplementary data files. Genome assemblies have been deposited at DDBJ/ENA/GenBank under accession numbers JAUTEI000000000–JAUTIF000000000 (see Table S1, available in the online version of this article), associated with BioProject PRJNA999611 (https://www.ncbi.nlm.nih.gov/bioproject/PRJNA999611).

## Introduction

*Streptococcus suis* is an emerging zoonotic pathogen, causing invasive infections and substantial economic losses in the pig industry worldwide. *S. suis *is classified in 29 serotypes on the basis of the antigenicity of capsular polysaccharides [1]. In Europe, serotypes 2, 7 and 9 are dominant among clinical pig isolates whereas, in humans, most clinical cases are associated with serotype 2 strains [2]. The nontypeable isolates that have been described harbour new variants of the *cps* locus [3,5]. Studying the population structure and the genetic diversity of *S. suis* is helpful to understand its epidemiology. Multilocus sequence typing (MLST) is a popular molecular typing method applied to study genetic diversity in *S. suis* [6]. It helps defining sequence types (STs) and clonal complexes (CCs) [7]. There are currently 2337 MLST profiles defined for *S. suis* (https://pubmlst.org/, last update 29 June 2023) that can be grouped in many CCs. The most important CCs causing infections in humans and pigs are CC1, CC16, CC20, CC25, CC28, CC94, CC104, CC233/379 and CC221/234 [6].

Several *S. suis* virulence factors have been characterized but it remains difficult to predict pathotypes based on the presence or absence of those factors because strains from different geographical areas seem to have different virulence profiles [8]. Several studies have been published on the virulence factors involved in the pathogenesis of American, Asian and some European strains [8,13] but data are missing for strains isolated in France.

In *S. suis*, antimicrobial resistance (AMR) is mainly conferred by acquired genes located on mobile genetic elements (MGEs), which are responsible for the dissemination between bacteria sharing the same environment [14,16]. Whole genome sequencing (WGS) combined with comparative genomic analyses was used to study AMR genes and their location on MGEs in *S. suis* strains isolated from various countries [12,15, 17, 18]. These studies showed that two categories of MGEs called integrative conjugative elements (ICEs) and integrative mobilizable elements (IMEs) play an essential role in AMR dissemination in *S. suis*. ICEs encode their own excision, transfer by conjugation and integration. In addition to the genes involved or controlling their mobility, all ICEs carry cargo genes which can provide properties (adaptation, virulence, antibiotic resistance) advantageous for their bacterial host [19,20]. IMEs also encode their own excision and integration in the bacterial chromosome but rely on the conjugative apparatus of conjugative elements to transfer by conjugation. These mobile elements have been less studied than ICEs because they are more difficult to identify in bacterial genomes, particularly in streptococci since they are frequently found as composite elements (accretion with other MGEs in tandem or integrated inside) [21,22].

*S. suis*, like several other streptococci (*Streptococcus thermophilus*, *Streptococcus pneumoniae*, *Streptococcus mutans*), is naturally competent i.e. can capture extracellular DNA and integrate this DNA in its chromosome [23,24]. This other mechanism of gene transfer can contribute to the acquisition of AMR genes (located on MGEs or not) from the surrounding bacterial environment. As described by Zaccaria *et al*. [25], *S. suis* needs several systems in order to incorporate extracellular DNA in its chromosome: components to produce a pheromone (ComS), for its processing (hypothesis of Eep protease involvement), import (Opp peptide transport system) and detection (thanks to the transcriptional regulator ComR) plus an alternative sigma factor (ComX), a DNA-binding pilus, a DNA transport system and a recombination synapse enabling integration of DNA in the chromosome. Three pherotypes, i.e. production of different pheromones, associated with different CCs, have been described in *S. suis* [26].

We recently described the antimicrobial susceptibilities of a collection of 200* S*. *suis* strains isolated from 1995 to 2020 in different regions of France from different hosts (humans, pigs and wild boars) [27]. WGS was done on half of these strains (93 resistant strains and nine strains susceptible to all antibiotics) representative of the diversity of serotypes observed in the collection in order to characterize (i) their genetic diversity, (ii) their virulence gene pattern, (iii) the genes conferring AMR in these isolates and the genetic environment of those genes, i.e. their location on MGEs, as well as (iv) their putative competence and pherotype.

## Methods

### Strains and growth culture

The collection of strains that was subjected to WGS include not only strains of the main serotypes found in clinical human or animal infections but also less frequent serotypes (serotypes 5, 10, 11, 12, 14, 16, 18, 27, 29) (Table S1).

Bacterial strains (*n*=102), stored at −80 °C in buffered peptone water solution with glycerol (20 %), were sub-cultured on Columbia Agar with 5 % Sheep Blood (Bio-Rad) and incubated overnight at 37 °C under 5 % CO_2_.

### DNA extraction and WGS

DNA extraction was performed with the NucleoMag Tissue kit from Macherey-Nagel and quantification of DNA was done with the Qubit ds DNA HS Assay kit from Invitrogen.

WGS was performed by the iGenSeq genotyping-sequencing platform of the Paris Brain Institute (https://institutducerveau-icm.org/en/igenseq-platform/) with an Illumina Novaseq 6000. The quality of the reads was checked using FastQC developed by S. Andrews (v0.11.9, available at https://www.bioinformatics.babraham.ac.uk/projects/fastqc/). The reads were cleaned with fastp (v0.20.1, options: ‘−5–3 --overrepresentation_analysis --fix_mgi_id --adapter_fasta oligo_file_illumina --detect_adapter_for_pe --correction’) [28]. *De novo* assemblies were carried out using the Shovill pipeline developed by T. Seemann (available at https://github.com/tseemann/shovill), with contigs of size higher than 200 nt and kmer coverage higher than 2. The quality of the assemblies was analysed using Quast (Quality Assessment Tool for Genome Assemblies) [29] and CheckM [30].

### Genomic analyses

MLST was done using the pipeline developed by Athey *et al*. [31]. MLST groups were attributed with the scheme developed by King *et al*. [32] using pubMLST [7] (https://pubmlst.org/). New allele sequences and STs were uploaded to PubMLST to obtain the allele profiles and define the genotypes. On the basis of their respective allele profiles, the *S. suis* isolates were clustered by using the eBURST program (available as a plugin for the BIGSdb database software at https://pubmlst.org/). When six of seven alleles among the *S. suis* isolates were identical, these isolates were grouped in the same cluster. Using this stringent group definition (6/7 shared alleles), isolates in a group defined by eBURST are considered to belong to the same CC [33,34]. The isolates that did not belong to any cluster were indicated as singletons.

A total of 70 genetic markers that can be associated with virulence (see list modified from Wileman *et al*. [35] in Table S2) was searched by local customBlast with the BlastX program (E-value cut off 1×10^−20^) using the Geneious prime software (v2023.2.1; Biomatters). A hit was retained only if it shows more than 70 >% amino acid identity with the query and at least 80 % coverage of the query.

Pseudochromosomes were generated by aligning each set of contigs to 21 reference complete genomes (see list in Table S3) using the MeDuSa draft genome scaffolder [36]. Remaining contigs were concatenated to the pseudochromosome using Geneious prime software (v2023.2.1; Biomatters). Pseudochromosomes were annotated with Prokka [37] using the reference strain BM407 (RefSeq accession number: GCF_000026745.1) and used as inputs for the recently described ICEscreen pipeline [38] (v0.4, https://icescreen.migale.inrae.fr/) in order to identify MGEs present in the genomes. Elements encoding the four signature proteins of ICEs (integrase, coupling protein, relaxase and VirB4) were counted as putative ICEs and those with at least one pseudogene as defective ICEs. Likewise, elements were considered as putative IMEs if they encode at least an integrase and a relaxase and as defective IMEs if one or both of these genes is/are a pseudogene.

Annotated genomes were used as inputs for analysis by Roary [39] to extract and make an alignment of core genes (using a BlastP 90 % threshold, the ‘don’t split paralogs’ option). The multi-FASTA alignment was then used to generate a phylogenetic tree of the strains using IQTree 2.2.0.3 [40] with the ModelFinderPlus option [41] in order to select the best-fit evolutionary model. One divergent clade was used as an outgroup to obtain an outgroup-rooted tree. Genomic relatedness of the strains was also analysed using dRep (determination of average nucleotide identity or ANI without primary clustering) [42].

The search for AMR genes was done by local customBlast with the BlastN program (E-value cut off 1×10^−20^) using the Geneious prime software (v2023.2.1; Biomatters), against the imported ResFinder 4.1 [43] and the CARD 3.0.3 database [44]. Other AMR variants previously described in *S. suis* [45,47] were searched (see Table S4). A hit was retained only if it shows more than >70 % nucleic acid identity with the query with at least 80 % coverage of the query. The genetic environment of the AMR genes was analysed using the Geneious prime software (v2023.2.1; Biomatters). We used the output annotated files generated by ICEscreen to see if the AMR genes were located on MGEs.

Modifications in the five penicillin binding proteins (PBPs), PBP 1a, PBP 1b, PBP 2a, PBP 2b and PBP 2x, in the acetylmuramoylpentapeptide-transferase MraY, and in the quinolone resistance determining region (QRDR) of the two sub-units of the gyrase (GyrA, GyrB) and the two sub-units of the topoisomerase IV (ParC and ParE) were analysed in the resistant isolates by comparing the protein sequences with those of closely related susceptible isolates and to the sequences of *S. suis* P1–7 used as reference. Alignments were made using Muscle 5.1 [48].

Competence was analysed by searching 38 genes (corresponding to the required components described above, with three different variants for ComR and ten different variants for ComS; see list in Table S5 derived from the literature [25,26]) in the genomes by local customBlast interrogation of a home-built database using the BlastX program (E-value cut off 1×10^−20^). Variants with less than 70 % amino acid identity with the query were manually checked and analysed for conserved domains and residues. A pherotype was attributed to the strains with a complete set of competence genes.

### Statistical analyses and data visualization

Due to the large number of genes analysed, principal component analysis (PCA) and hierarchical clustering on principal components (HCPC) analysis were used to identify the most discriminatory genetic markers. To decrease the number of modalities per qualitative variable, the less frequent ones were grouped (for serotypes and CCs). For quantitative variables (number of ICEs, number of IMEs, number of ICEs+IMEs, number of AMR genes, number of virulence markers and genome length), we calculated the median and quartiles and defined three groups: <quartile (Q)1, in interquartile range (IQR), >Q3. Chi-square tests were used to analyse the correlations between 12 variables: ‘year’ (two modalities: ‘before_2010’ and ‘after_2010’), ‘pathotype’ (four modalities: carriage, respiratory, systemic or unknown), ‘serotype_group’ (seven modalities: 1, 1_2, 2, 3, 7, 9 or other), ‘CC_group’ (six modalities: CC1, CC16, CC28, CC231, other or singleton), ‘pherotype’ (four modalities: I, II, III or NA), ‘virulence_cluster’(three modalities: cluster_1, cluster_2 or cluster_3) and six variables with three modalities (<Q1, in IQR and >Q3): ‘ICE_total_group’, ‘IME_total_group’, ‘ICE+IME_total_group’, ‘AMR_total_group’, ‘virulence_total_group’ and ‘genome_length_group’. Only results with a *P*-value ≤0.01 (i.e. 99 % level of confidence) were considered significant. PCA, HCPC analysis and Chi-square tests (with Fisher correction for limited expected counts) were done using R [FactoMineR 2.8 package withthe PCA and HCPC functions and chisq.test (fisher.test) respectively].

The integration and visualization of the genomic and statistical data were done using the ggplot2 3.4.2 [49], UpsetR 1.4.0 [50] and ComplexHeatmap 2.16.0 [51] packages in R.

## Results and discussion

The 102 genomes passed the various steps of quality control (see Methods and Table S6 for quality data for each genome). This set comprises the genome of 91, eight and three strains isolated from pigs, humans and wild boars respectively (Table S1).

### Genome diversity of 102 French isolates of *S. suis* isolated from pigs, humans and wild boars

Genome length of the 102* S*. *suis* strains was between 1 966 158 and 2402398 bp (IQR=2046355–2204208, median=2 103 666, Table S6).

**Fig. 1.**
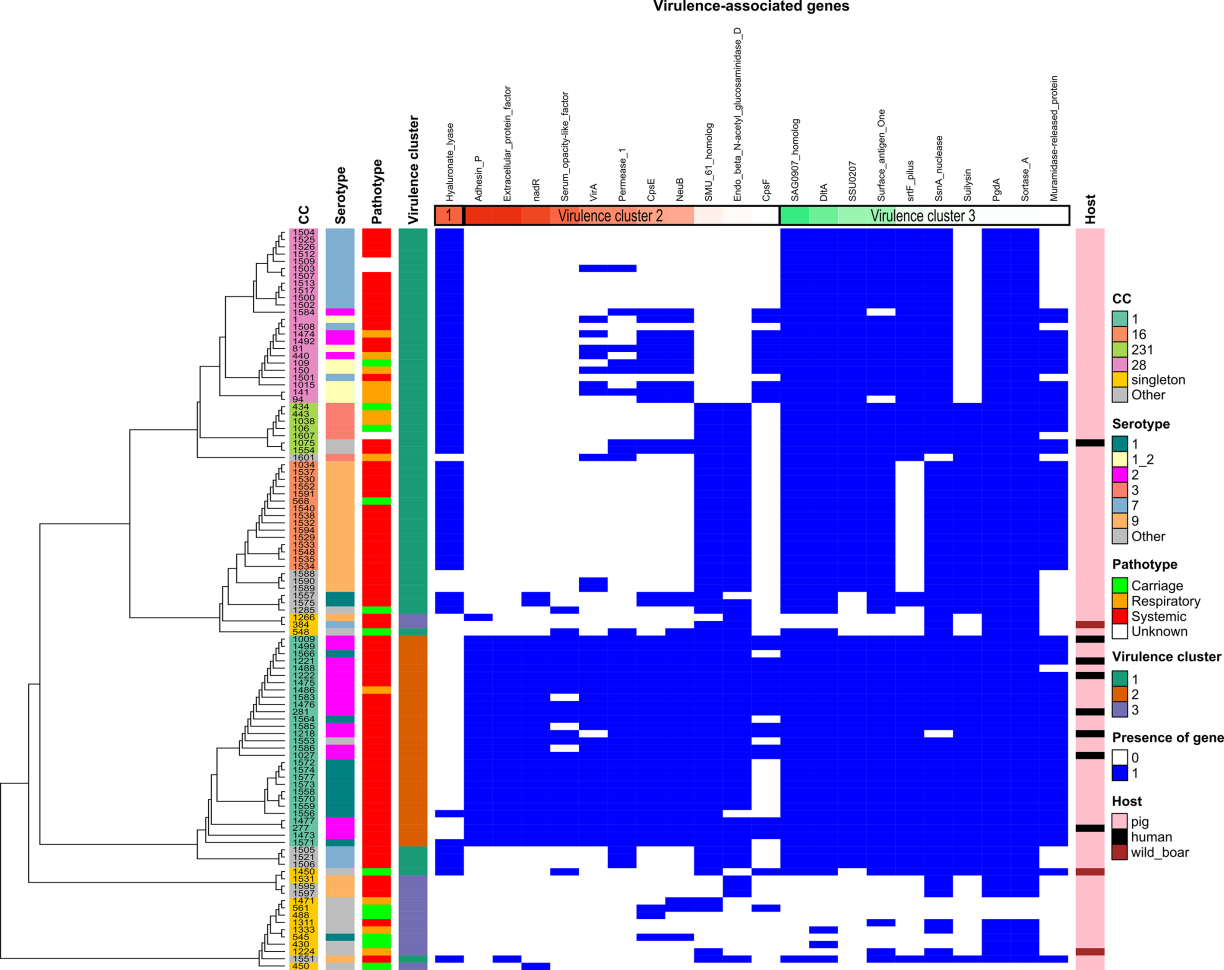
Heatmaps of (**i**) clonal complex (CC), (ii) serotype, (iii) pathotype, (iv) virulence-gene pattern and (iv) host for the 102 genomes of French isolates of *Streptococcus suis*. Genomes have been grouped according to their phylogenetic distance determined by alignment of their core genes as indicated by a phylogenetic tree at the left (see Methods). The names of the strains are indicated in the first column (CC heatmap). Columns 2 and 3 indicate the serotype and pathotype of the strains respectively. Column 4 gives the virulence cluster of the strain obtained by HCPC analysis (see Methods). The grouping of the strains into three virulence clusters was made according to the presence (in blue) or absence (in white) of 22 discriminant virulence-associated genes among the 70 genes analysed: one gene characteristic of cluster 1 (shown in column 5, box ‘1’), 11 genes characteristic of cluster 2 (columns 6–16 inside a ‘virulence cluster 2’ box) and ten genes characteristic of cluster 3 (columns 17–26 inside a ‘virulence cluster 3’ box). The 22 genes are ordered according to their discriminatory power (by descending v.test values obtained by HCPC with a positive value, i.e presence of the gene, in brown and a negative value, i.e absence of the gene, in green). Column 27 indicates the host of the strains.The colour code of each column is shown on the right side of the figure.

MLST of the 102 strains led to the identification of 31 new STs (1505–1509, 1514–1523 and 1854–1869) in our collection. The most prevalent STs were ST1 (*n*=29), followed by ST16 (*n*=14), ST28 (*n*=11), ST29 (*n*=11), ST231 (*n*=6), ST1505 (*n*=3), 1508 (*n*=3), ST13 (*n*=2), and ST94, ST1515, ST1516, ST1518, ST1519, ST1521, ST1522, and ST1854–ST1869 (*n*=1 for each ST group). Most of the strains (*n*=88) could be clustered in CCs on the basis of their MLST alleles. Eleven CCs were identified, the most frequent being CC1 (*n*=29), CC28 (*n*=24) and CC16 (*n*=15) (Fig. 1, Table S1).

Comparison of genomes using Roary identified 1144 genes shared by more than 95 % of the strains (‘core genes’ and ‘soft core genes’). This is close to the theoretical 980 core genes calculated by Chen *et al*. [52]. Alignment of these core genes was used to build the phylogenetic tree shown in Fig. 1. Clusters obtained by this method are in accordance with the CCs defined by MLST.

The genomic relatedness of the strains was also evaluated by calculating the ANI between strains. Four strains (450, 488, 561 and 1471) appear very distant from the others. Three of them are carriage strains from pigs (one nasal carriage and two isolated from tonsil) isolated before 2010 and the fourth was isolated from lungs of a pig after 2020 (Table S1). These strains were attributed a new ST (1854, 1857, 1858 and 1861). These divergent strains exhibit 89–90 % ANI with the other genomes compared to the standard 95 % ANI species-level cutoff [53]. Such diversity inside *S. suis* has already been described [54,55].

Most of the strains of serotype 2 belong to ST1 (17 ST1 and four ST28) as reported in other European countries [2]. Interestingly, strains with the same ST exhibit different serotypes: ST1 strains (CC1) with serotypes 1, 2 and 14; and ST231 strains (CC231) with serotypes 3 and 14 (Fig. S2, Table S1). Since the serotype of the strains was confirmed using reference antisera [27], this indicates capsular switching in these strains. Intraspecies horizontal transfer of serotype-specific *cps* genes has already been suggested [56]. Switching from serotype 2 to serotype 14 was reported to have no effect on virulence for the ST1 strain P1/7 [57]. As far as we know, the impact of switching from serotype 3 to serotype 14 for ST231 strains has not been documented yet.

### Distribution of putative virulence genes among 102 French isolates of *S. suis*

**Fig. 2.**
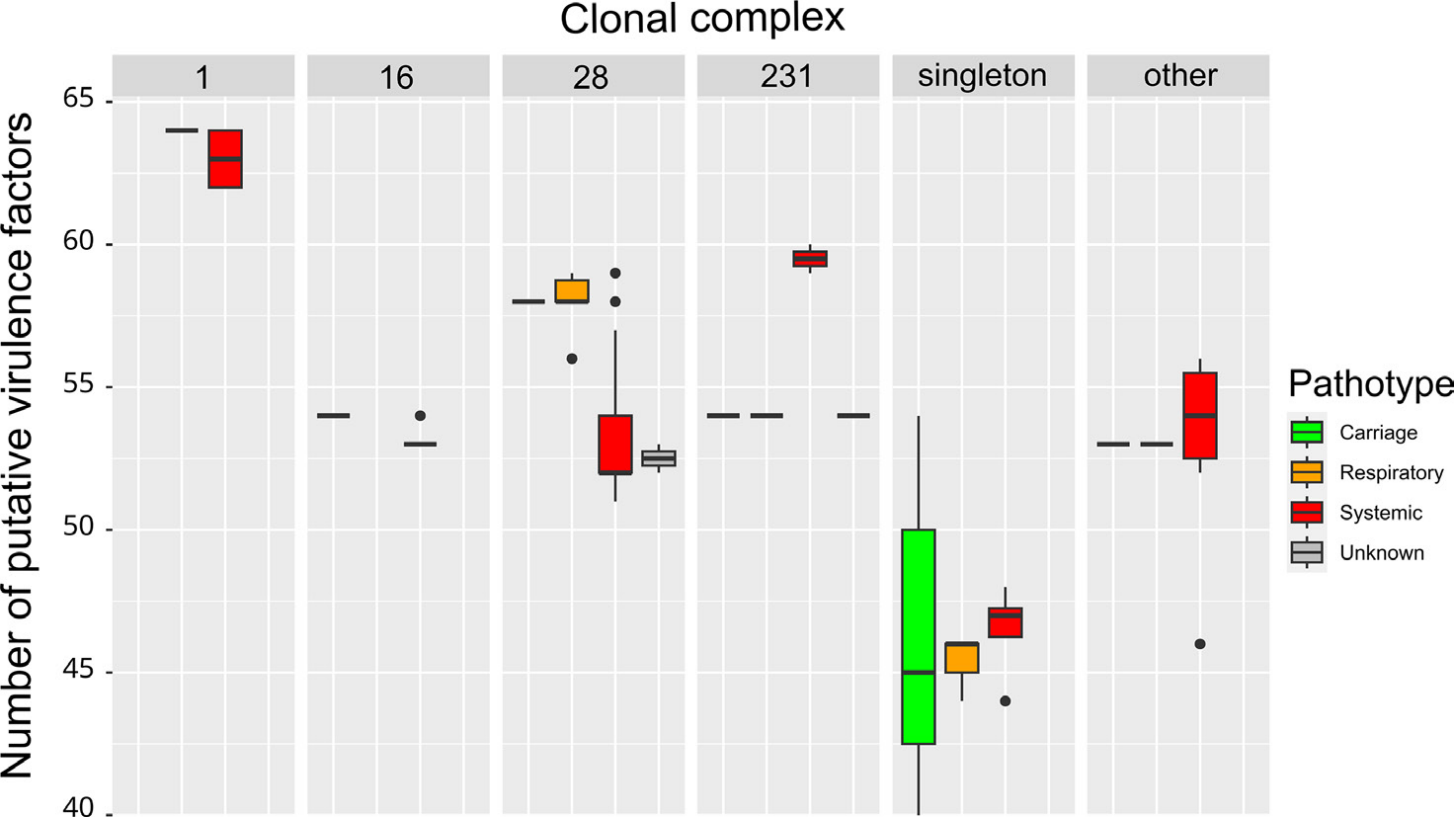
Number of putative virulence genes according to the clonal complex and the pathotype of the strains. Boxplots indicate the median value and the interquartile range (Q1–Q3. i.e. 25–75 % of the values). CCs with fewer than five strains were grouped in the category ‘other’.

The set of 102 genomes was screened for the presence of 70 virulence-associated markers described in *S. suis* (see Methods). Two markers (Lpp lipoprotein and RevS) were absent from the genomes and two were found in only three genomes (SalK and SalR). Thirty-nine markers were found in all or almost all the genomes. The other markers are found in 13–99 genomes (median=54 with IQR, i.e 25–75 % range of the values, =52–62) (Fig. 2 and Table S1).

HCPC analysis enabled the clustering of genomes in three clusters according to the presence/absence of the 22 most discriminatory genetic markers (Fig. 1). The most discriminatory marker of cluster_1 is hyaluronate lyase, whereas 11 markers (the most discriminatory ones being adhesin P, extracellular protein factor and NadR) are positively associated with cluster_2 (Fig. 1). Cluster_3 is characterized by the absence of some virulence-associated factors (the four most discriminatory being SAG0907 homologue, DltA, SSU0207 and surface antigen 1). Statistical analysis (chi-square tests with Fisher correction, see Methods) indicated a significant link between these clusters and (i) CCs (χ²=173.29, *P*<2×10^−16^) and (ii) the number of putative virulence-associated factors (χ²=137.22, *P*<2×10^−16^) but also (iii) with pathotypes (χ²=19.699, *P*=0.002). By contrast, there is no significant link between these clusters and the number of AMR genes (*P*>0.01). More precisely, strains of CC1, CC16, CC28 and CC231 are associated with specific patterns of virulence-associated factors. All CC1 strains are associated with cluster_2 whereas CC16, CC28 and CC231 are all associated with cluster_1. Cluster_2 is characterized by a higher number of putative colonization/virulence factors (100 % of the genomes belonging to the group ‘superior to Q3’ defined by more than 62 putative virulence genes are included in cluster_2 of virulence) and is preferentially associated with the pathotype ‘systemic’ (96.6 % of strains in cluster_2 are of pathotype ‘systemic’). By contrast, cluster_3 is characterized by fewer colonization/virulence factors (82.4 % of strains with fewer than 52 putative colonization/virulence factors belong to cluster_3). These associations appear clearly on Fig. 1. There is also a significant link between CC_class and the number of virulence factors (χ²=113.19, *P*=0.0005). In particular, CC1 strains show a higher number of colonization/virulence factors (all the genomes belonging to the group ‘superior to Q3’ for the number of virulence genes belong to CC1), as visible on Fig. 2. We also studied the correlation between the length of the genome and other variables. Chi-square tests indicated a link between the length of the genome and CC_class (χ²=78.476, *P*=0.0005), with a high proportion (62 %) of CC1 strains having a small genome. Statistical analyses also indicated a correlation between the length of the genome and the cluster of virulence (χ²=45.047, *P*=3.0×10^−9^), with a smaller length of genome associated with Cluster_2 of virulence (69.2 % of strains of cluster_2 have a smaller genome versus 26.9 and 3.8 % for cluster_1 and cluster_3 respectively) and a higher length of genome associated with Cluster_3 of virulence (71.4 % of strains of cluster_3 have a larger genome versus 27.1 and 0.0 % for other clusters respectively). A significant link was also observed between the length of the genome and the virulence gene content (χ²=33.182, *P*=2.0×10^−6^), with a smaller genome size being associated with a higher content of virulence genes (50 % of the smaller genome group have a higher number of virulence genes in comparison with 7.7 % with a smaller number of virulence markers). In addition, a significant link was found between genome length and pathotype (χ²=18.452, *P*=0.002), with 92.3 % of strains with smaller genomes having pathotype ‘systemic’ and 66.7 % of carriage strains having a genome of larger size. This indicates that the most pathogenic strains have a smaller genome due to genome reduction as previously reported by Weinert *et al*. [58] and that carriage strains expand their genome in order to adapt to changing conditions in their environment.

Through HCPC analysis, hyaluronate lyase was identified as a marker that distinguishes strains of CC1 from the other strains. Indeed, all but two strains (strains 1556 and 1571) of this CC are devoid of this factor. This is in accordance with previous studies indicating that many virulent strains of *S. suis* lack hyaluronate lyase activity [59]. This suggests that hyaluronate lyase is not an important virulence factor of *S. suis*.

The extracellular protein factor (EPF), which is one of the discriminatory markers of cluster 2 obtained by the HCPC analysis, is one of three virulence-associated factors, with the muramidase-released protein (MRP) and the suilysin, that have been extensively used to predict the virulence potential of *S. suis* strains mostly for strains of serotype 2 [8,60]. In the strain collection examined in this work, all the strains of CC1 (*n*=29), regardless of their serotype (1, 2 or 14), harbour the genes encoding these three factors (except strain 1488 that is *mrp*^−^) (Fig. 1, Table S1). This is not true for strains of these serotypes (1, 2 and 14) that belong to other CCs (which are all *epf*^−^). The *epf*^+^ *mrp*^+^ *sly*^+^ genotype appears predictive of ST1. Only one strain with this genotype belongs to another ST (strain 1551 with ST1521). Scherrer *et al*. [13] previously reported a link between the presence of these three virulence-associated factors and ST rather than with *cps* types. All the strains of CC1 with serotype 2, even the human isolates, encode the reference P1/7 EPF (called the ‘110 kDa variant’). In contrast to strains of CC1, strains of CC16 (*n*=15, all of serotype 9) are all *epf*^−^. This is in accordance with the observations of Wisselink *et al*. made on *S. suis* strains isolated from diseased pigs in seven European countries including France [60]. All the strains of CC16 are *epf*^−^ but *mrp*^+^ *sly*^+^. Only four of these strains encode a variant of MRP with a higher molecular weight (>136 kDa). This is in contrast with the observation of Wisselink *et al*. [60] who reported a majority of high-molecular-weight MRP in strains of serotype 9. In addition, half of the strains of CC28 are *epf^−^mrp^−^sly*^−^ (strains of serotype 7) while the other half are *mrp*^+^ (strains of serotypes 1–2 and 2). This is in accordance with a previous study made on French isolates of *S. suis* [61].

Two other invasive disease-associated markers (SSU0207 and SSU1589/VirA) were proposed by Wileman *et al*. [35]. These two factors are among the markers that enabled clustering of the strains through our HCPC analysis. As previously described by Scherrer *et al*. [13], VirA is present in all the strains (except one) of CC1 (virulence cluster_2) but is absent in carriage strains. Other *virA*^+^ strains belong to CC14 (serotype 9) (*n*=2), CC28 (serotypes 1–2, 2 or 7) (*n*=8) and CC94 (serotype 3). The absence of SSU0207 is characteristic of strains of cluster_3. This factor is present in most of the strains of cluster_1 (53/59) but not only in invasive strains since it was found in four carriage strains. We analysed the SSU0207 sequence of strains of CC28 to see if the encoded protein is full-length or with partial deletions as described by Scherrer *et al*. [13]. No deletion was found in the sequences analysed.

Thirty-six virulence-associated factors were found in the four divergent strains (Table S1), 23 of them being previously described in the divergent strains analysed by Baig *et al*. [54]. Most of them are metabolism-related. Most (17/22) of the discriminatory virulence-associated factors found by HCPC analysis are absent in these divergent strains (Fig. 1 and Table S1).

It should be pointed out that one strain of CC1 (strain 1486) was isolated from lungs of a pig (pathotype ‘respiratory’ and not ‘systemic’) but harbours the highest number of virulence-associated factors found in the genomes examined (64 markers in total) (Figs1  2, Table S1). Likewise, one strain of CC16 (strain 568), which is among the three strains of this CC that harbour the highest number of virulence-associated factors (54 markers in total), was isolated from the nasal cavity of a pig (pathotype ‘respiratory’ and not ‘systemic’). This suggests that these strains could have a high invasive potential even if they were isolated from the upper and lower respiratory tract of a pig.

### Prevalence and diversity of AMR determinants

**Fig. 3.**
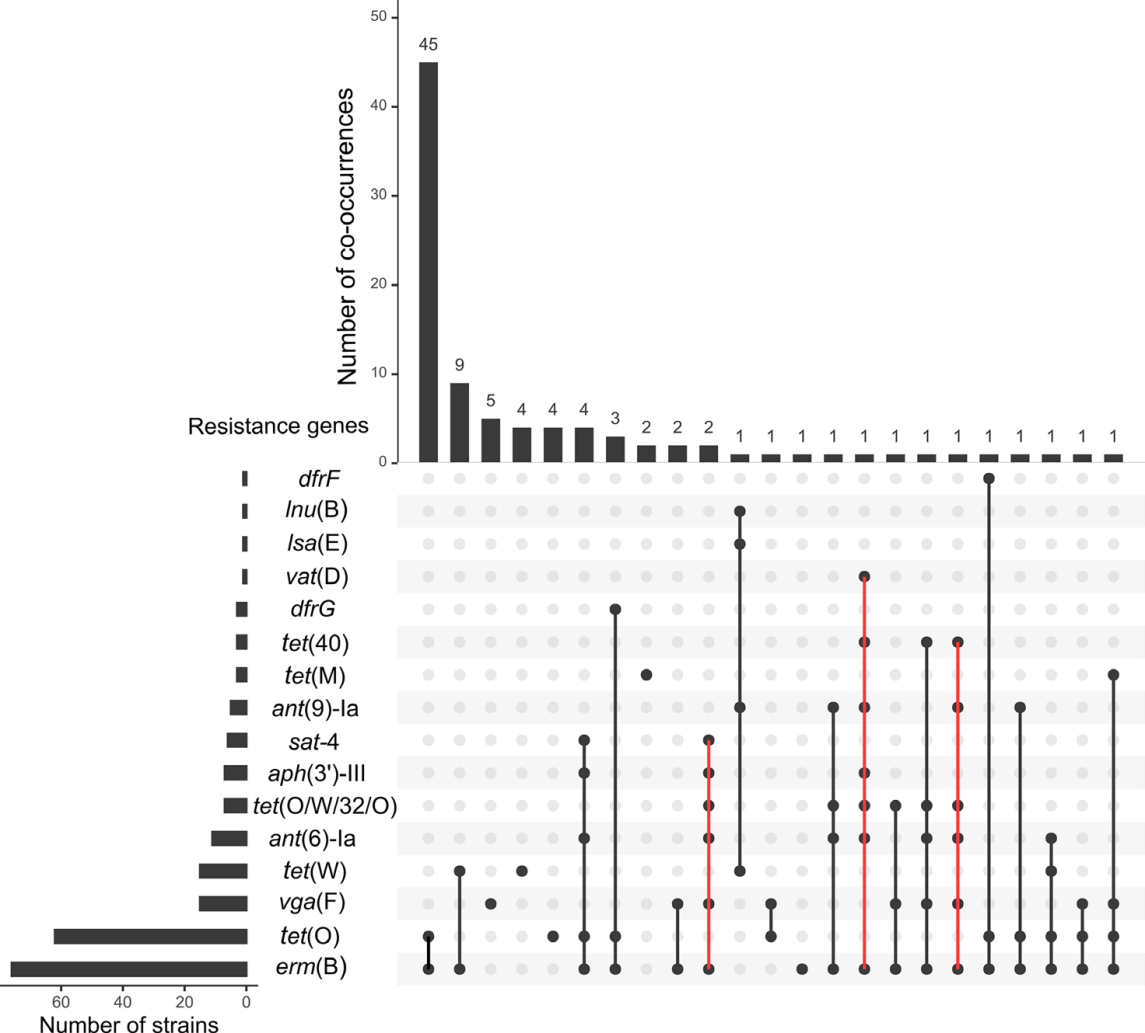
Prevalence and co-occurrence of antimicrobial resistance genes in the genomes of French isolates of *S. suis* (associations of more than six AMR genes are indicated in red).

We confirmed that the nine susceptible strains were devoid of AMR genes (Table S1). Among the 93 other strains, 217 putative AMR determinants were identified, corresponding to 16 different AMR genes (Figs 3 and S3, Table S1). As expected, the most prevalent genes are genes conferring resistance to macrolides and/or lincosamides and/or streptogramins [*erm*(B), *n*=76; *lnu*(B), *n*=1; *lsa*(E), *n*=1, *vat*(D), *n*=1] or tetracyclines [*tet*(O), *n*=62]. Four other tetracycline resistance genes were identified [*tet*(W), *n*=15; *tet*(O/W/32/O), *n*=7; *tet*(M), *n*=3; and *tet*(40), *n*=3]. The two most frequent associations of AMR genes are *erm*(B)*–tet*(O) (*n*=57) and *erm*(B)*–tet*(W) (*n*=10). Genes that were previously associated with resistance or reduced susceptibility to four other families of antibiotics were also detected: (i) aminoglycosides: *ant*(6)-Ia (*n*=11), *aph*(3′)-III (*n*=7) and *ant*(9)-Ia (*n*=5); (ii) streptothricin: SAT-4 (*n*=6); (iii) trimethoprim: *dfrG* (*n*=3) and *dfrF* (*n*=1); and (iv) pleuromutilin: *vga*(F) (*n*=15). Some strains harbour six or even seven AMR determinants (that can confer resistance to four to five different families of antibiotics) (Figs 3, S3 and Table S1).

All the strains that are resistant to macrolides (erythromycin, tylosin and tilmicosin) carry an *erm*(B) resistance gene (*n*=76). Only one strain resistant to lincosamides (lincomycin/clindamycin) but susceptible to macrolides carries *lnu*(B) and *lsa*(E) resistance genes. The four other strains resistant to lincosamides carry a gene with 86–88 % identity *to vga*(F) described by Hadjirin *et al*. [45]. Further analysis of these genes indicated that they encode an ABC-F protein with 84–86 % identity to the recently described ribosome protection protein SrpA [62]. This determinant was shown to confer not only resistance to pleuromutilin but also to lincosamides. All but one of the strains resistant to tiamulin (*n*=11) carry this *srpA* gene homologue. Thus, this resistance determinant appears widespread in pig isolates of *S. suis* in France and probably confers resistance to several families of antibiotics. Interestingly, it was also detected in two wild-boar isolates. However, we did not detect any Insertion Sequence (IS) sequence upstream and downstream of the resistance gene that could enable formation of a circular form as described by Zhang *et al*. [62]. However, interestingly, in one strain (strain 1551), the *srpA* gene is interrupted by a genetic element that encodes a MobQ relaxase (with a PF03389 domain). There is no evidence that this element is mobile since it does not carry any integrase gene. Among the 90 strains that are resistant to tetracycline, five (including three of serotype 9) do not carry any detectable tetracycline resistance gene. In addition, only four genes conferring resistance to trimethoprim (three *dfrG* and one *dfrF*) have been detected. We did not identify candidate genes in the 18 other strains with this phenotype of resistance (Table S1).

**Fig. 4.**
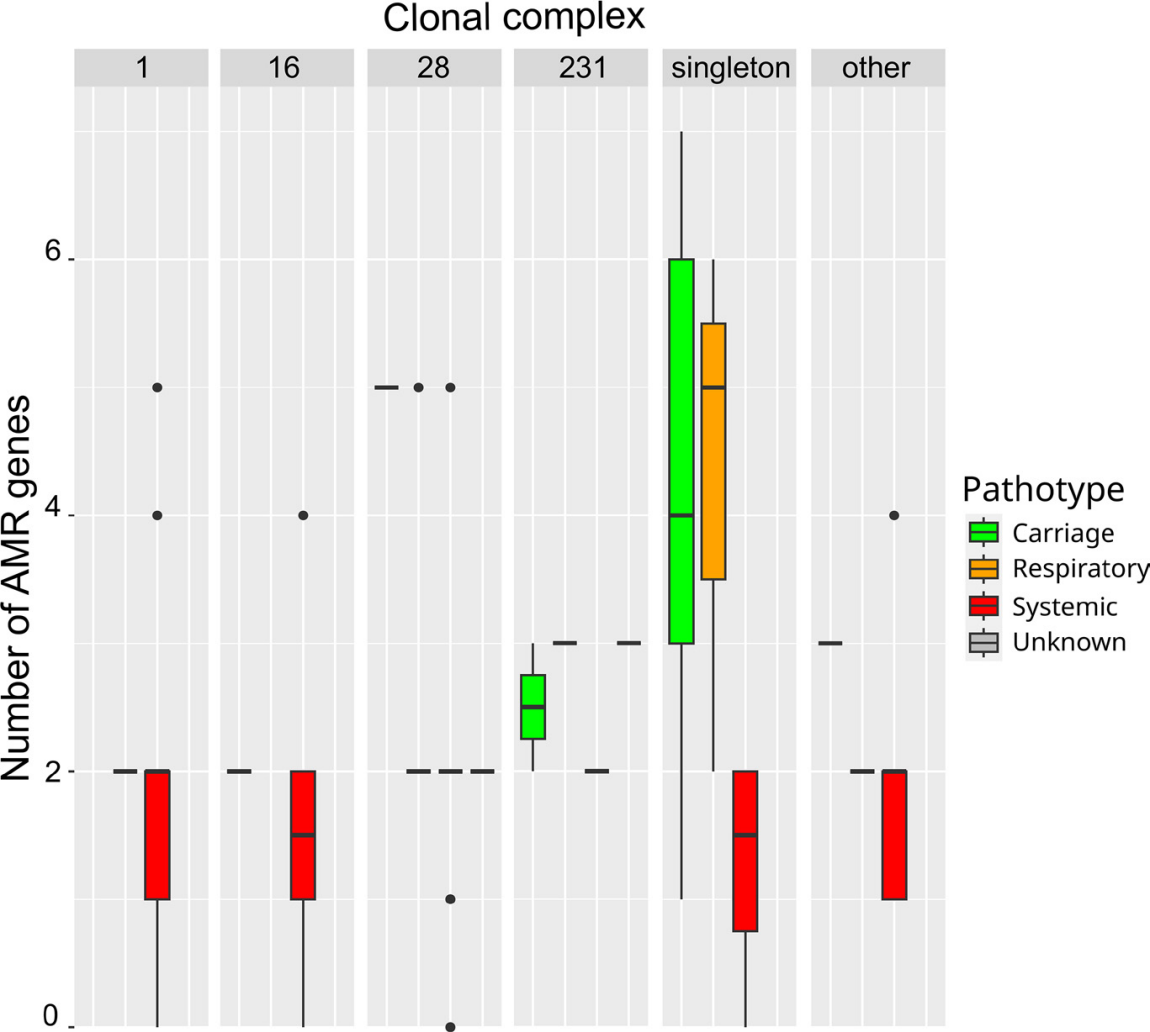
Number of AMR genes according to the clonal complex and the pathotype of the strains. Boxplots indicate the median value and the interquartile range (Q1–Q3, i.e. 25–75 % of the values).

Statistical analysis (chi-square tests) indicated a significant link between AMR gene content and pathotype (χ²=30.641, *P*=1×10^−5^), with the pathotype ‘systemic’ showing a lower content of AMR genes (96 % of strains of the ‘<Q1 group’, i.e. fewer than two AMR genes, have a pathotype ‘systemic’) and the ‘carriage’ pathotype preferentially associated with a higher content of AMR genes (66.7 % of the carriage strains have a higher number of AMR genes) (Fig. 4). This is consistent with higher opportunities for gene exchanges in carriage strains than in invasive strains. In addition, a significant link was observed between AMR gene content and the isolation year of the strains (χ²=16.087, *P*=0.002), with 46.2 % of the strains isolated before 2010 having a higher AMR gene content versus only 10.5 % of the strains isolated after 2010 (Table S1). This suggests that the French Ecoantibio plan had an impact on the prevalence of AMR genes in *S. suis*.

We analysed the sequences of the five PBPs (PBP 1a, PBP 1b, PBP 2a, PBP 2b and PBP 2x) described in *S. suis* and of the acetylmuramoylpentapeptide-transferase MraY in the nine penicillin-resistant isolates identified in our strain collection (marked by a star in Fig. S1). Comparison with the protein sequences of closely related susceptible isolates indicated modifications in three PBPs, namely PBP 1a, PBP 2b and PBP 2x, that could be responsible for the resistance phenotype. Six major modifications were detected in the transpeptidase domain of PBP 1a (Pro409Thr, Lys522Glu/Gln, Lys525Arg/Gln, Asn459Ala/Asp, Ser477Gly/Asp and Ser578Ala/Asn/Lys found respectively in nine, eight, eight, seven, seven and six of the nine penicillin-resistant isolates) (Fig. S4). All these PBP 1a modifications have been described previously [63,64]. Many modifications were detected in the transpeptidase domain of PBP 2b (Fig. S5). All except one (Ser589Ala) have already been described in penicillin-resistant isolates of *S. suis* [45,63]. Many modifications were also detected in the transpeptidase domain of PBP 2x (Fig. S6). Most of them (except Ala460Val, Thr491Ser and Arg514Asp/Gly) have already been described previously in penicillin-resistant isolates of * S. suis* [45,63,65]. Two modifications (Met144Ile/Leu/Thr and Val145Ile/Leu) were detected in the glycosyltransferase domain of MraY. Five resistant isolates exhibited both modifications (Fig. S7). To our knowledge, modifications of MraY at these positions have not been reported until now.

We also analysed the QRDR of GyrA, GyrB, ParC and ParE of the multi-drug-resistant *S. suis* pig isolate 1471 (marked by a # in Fig. S1). GyrA Ser81Ala and ParC Ser79Ala modifications were detected in this fluoroquinolone-resistant isolate (Fig. S8). These modifications are classically observed in fluoroquinolone-resistant isolates of *S. suis* [66,70] but also in other bacterial species [71]. No modification was observed at position 85 of GyrA. Interestingly, a GyrA Ser81Gly modification was observed in one fluoroquinolone-susceptible isolate (1311) without modification in ParC. Both observations contradict the ParC79→GyrA85→GyrA81 mutation path proposed for *S. suis* [70]. No modification was observed in GyrB or ParE (data not shown).

### Analysis of ICEs and IMEs in the set of 102 genomes of French isolates of *S. suis*

#### Distribution of ICEs and IMEs in the analysed genomes

Among the 102 genomes studied, 129 (+21 defective) ICEs and 167 (+55 defective) IMEs were detected (Table S1). Strains host up to eight ICEs/IMEs and only one strain (isolate 1556) is devoid of ICEs and IMEs. Half of the strains host three to five integrative elements.

**Fig. 5.**
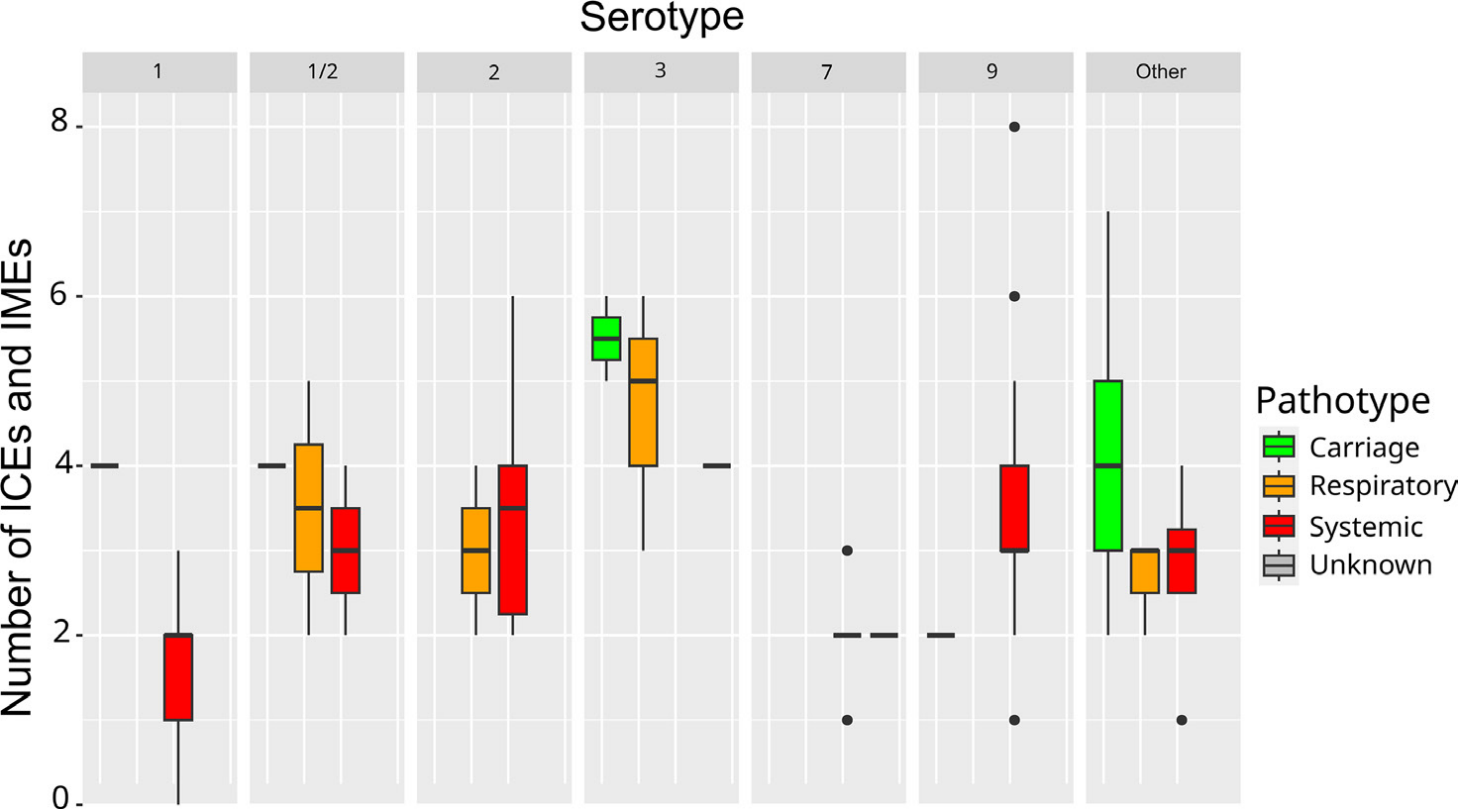
Number of ICEs and IMEs according to the serotype and the pathotype of the strains. Boxplots indicate the median value and the interquartile range (Q1–Q3, i.e. 25–75 % of the values).

If we consider the distribution of ICEs and IMEs according to the serotype of the strains, a significant link was noted between ICE/IME content and serotype (χ²=41.151, *P*=0.0005), with strains of serotype 1 and serotype 7 harbouring fewer elements than the other serotypes (85.7 and 82.4 % of the strains in the ‘<Q1 group’ for ICE/IME content respectively) (Fig. 5).

#### Diversity of ICEs and IMEs in the genomes

**Fig. 6.**
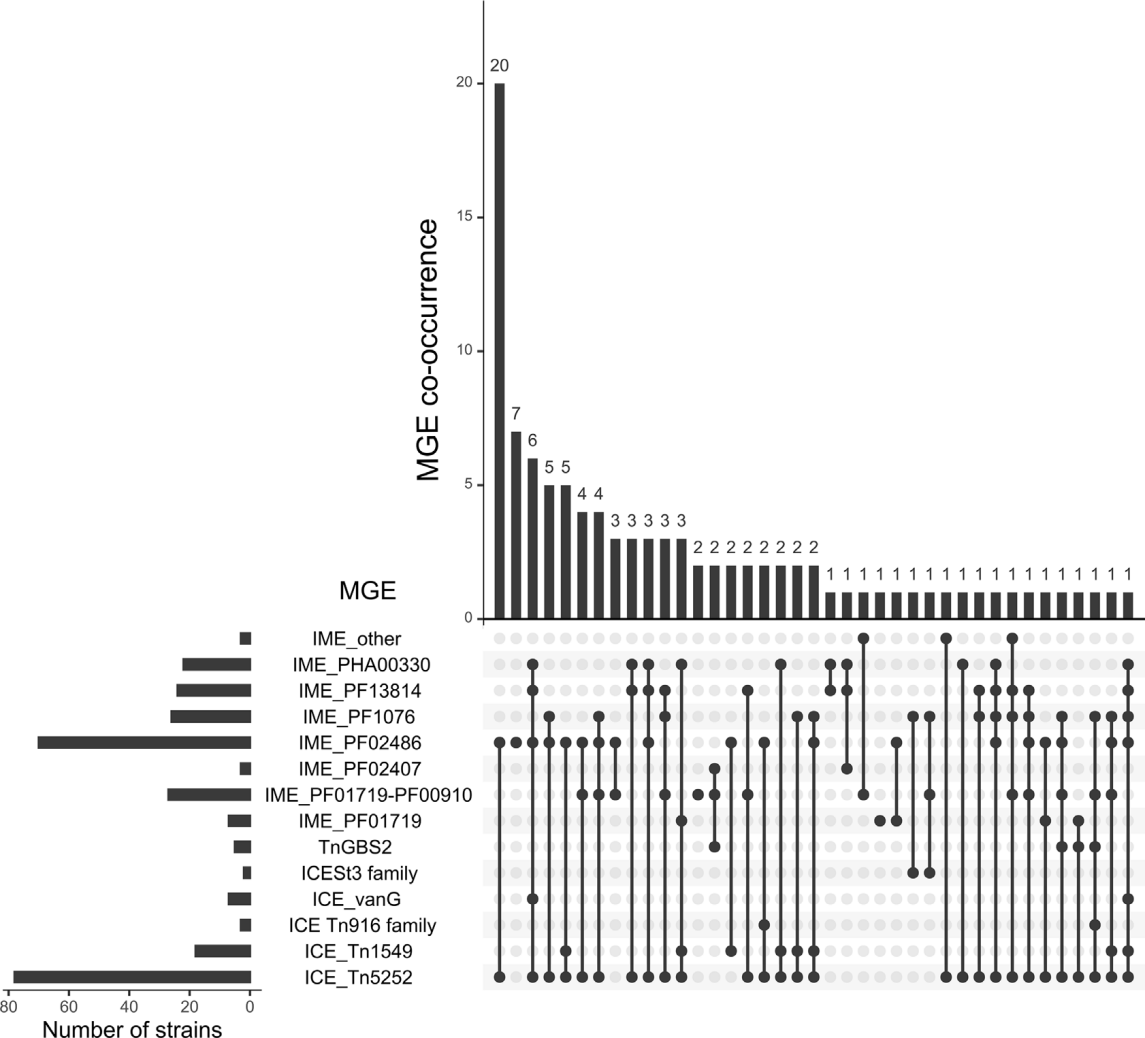
Prevalence and co-occurrence of ICEs and IMEs in the genomes of French isolates of *S. suis*.

ICEs found in the 102 genomes belong to six families according to their conjugation module (as described in other streptococci [72]): Tn*5252* (*n*=94), Tn*1549* (*n*=18), Tn*916* (*n*=3), ICE_*vanG* (*n*=7), ICE*St3* (*n*=2) and Tn*GBS2* (*n*=5) families (Fig. 6). Some strains host up to three different ICEs of the Tn*5252* family (Table S1).

IMEs can be classified according to the family of their relaxase [22]. The most prevalent family of IMEs detected in the genomes harbours a relaxase with a PF02486 domain (MobT family, *n*=72) (Fig. 6). Most of them are integrated in a tRNALeu gene (*n*=68) (Table S1). This was also one of the most prevalent categories of IMEs in a previous study conducted on Canadian isolates of *S. suis* [15]. Most of the defective IMEs (*n*=50) are integrated inside a gene of an ICE of the Tn*5252* family. In most cases, their integrase gene is interrupted by the insertion of a genetic element carrying an *erm*(B) gene.

#### ICEs and IMEs carrying AMR genes

**Fig. 7.**
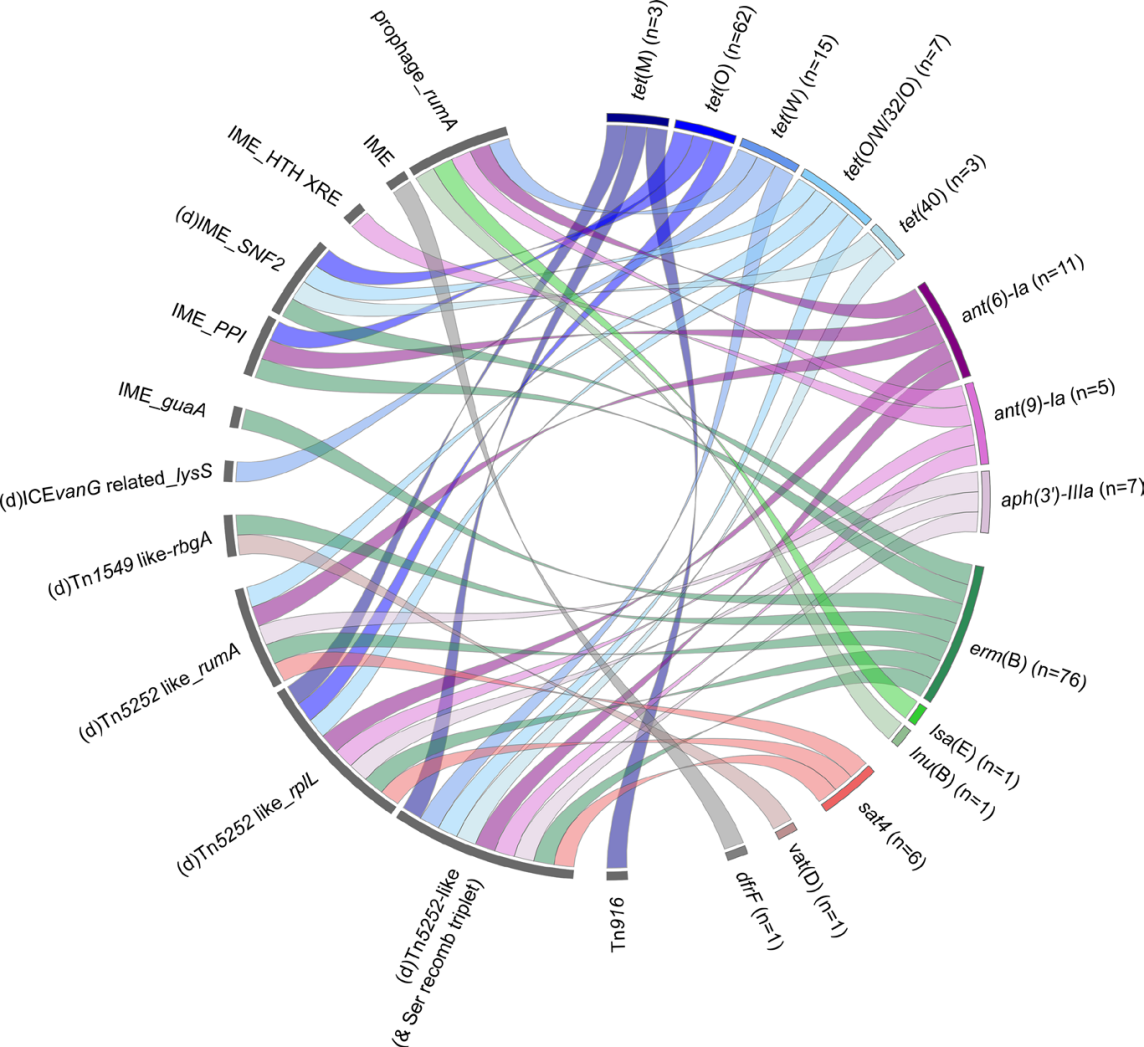
Mobile genetic elements carrying AMR genes in the genomes of French isolates of *S. suis*. Ribbons indicate the association between resistance genes (appearing in the right panel) and mobile genetic elements (in the left panel). Genes conferring resistance to the same families of antibiotics are grouped and indicated in the same colour (blue for tetracyclines, purple/pink for aminoglycosides, green for macrolides/lincosamides, red for streptothricin, brown for streptogramin A and grey for trimethoprim). The total number of AMR genes detected in the genomes is indicated after the name of each gene (this number includes all the genes, including those whose location was not determined due to gaps in the draft genomes).

Among the 217 AMR genes identified, 186 were found to be located on an MGE (Fig. 7 and Table S1). Information is lacking for 13 AMR genes due to their location on short contigs. AMR genes are mostly localized on IMEs (*n*=18) or defective IMEs (*n*=91). To our knowledge, several IME–AMR gene combinations are reported here for the first time: IME_*SNF2–tet*(O/W/32/O), IME_PPI*–ant*(6)-Ia, IME_HTH-XRE*–ant*(9)-Ia, IME_*guaA–erm*(B) and an IME with a MobT relaxase carrying a *dfrF* gene. All of these IMEs carrying an AMR gene, except two (IME integrated in a *guaA* gene and IME integrated in a gene encoding an HTH-XRE regulator), are themselves located on an MGE (106 on an ICE or defective ICE and one on a prophage). In all the cases, the ICE that hosts an IME/dIME belongs to the Tn*5252* family and the integration occurs into a *SNF2* gene or into a *PPI* gene. The most commonly found combination of AMR genes was *erm*(B)*–tet*(O) carried by an IME/dIME inserted in *SNF2* of an ICE of the Tn*5252* family (*n*=41). This high frequency of *erm*(B)*–tet*(O) association has been extensively reported in *S. suis* [12,16, 17, 45]. In addition to these AMR genes found on nested elements, 72 additional AMR genes were located on ICEs (seven of the *vanG* family, three of the Tn*916* family and one of the Tn*1549* family). This is the first report of a *vat*(D) on an ICE of the Tn*1549* family. Until now, this gene has been described only on plasmids (in *Enterococcus faecium*) [73] and on a phage in * S. suis* [12]. Finally, five AMR genes [one *ant*(6)-Ia, one *ant*(9)-Ia, one *tet*(W), one *lnu*(B) and one *lsa*(E) gene] were located on a prophage (Fig. 7 and Table S1).

### Competence genes

We evaluated if the strains harbour the whole set of genes required for acquisition of extracellular DNA by transformation. Most of the strains (*n*=95) harbour the whole set of genes (Table S1). This is interesting since this suggests that horizontal transfer of AMR genes by natural transformation could be widespread in *S. suis*. Experiments with plasmid and linear DNA carrying resistance genes made by Zaccaria *et al*. [23] indicated that *S. suis* can reach a high level of natural competence with an efficient uptake mechanism. Yu *et al*. [46] recently succeeded in transferring a genomic island carrying *tet*(L), *tet*(M) and *catA8* genes conferring tetracycline and chloramphenicol resistances from *S. suis* SC128 to *S. suis* P1-7. In addition, recent studies have uncovered a link between competence and the production of bacteriocins in streptococci. Exploitation of bacteriocin-mediated killing during competence provides adaptive gain to the bacteria [74].

**Fig. 8.**
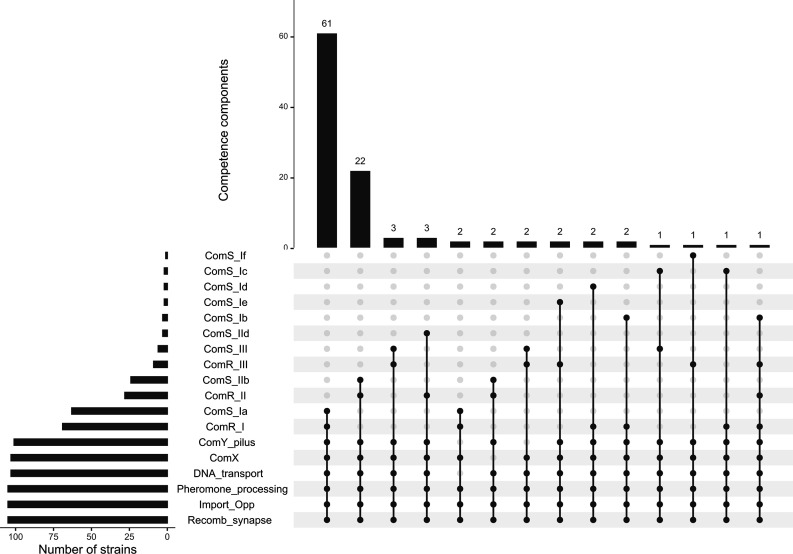
Distribution of the *com*RS variants/pherotypes in the genomes of French isolates of *S. suis*.

Several strains (*n*=6) harboured two copies of the *comX* gene. Truncations were observed in: the *comR* gene (*n*=1), *comX* (*n*=2), *comYA* (encoding a minor pilin, *n*=2) and *comYA-comYG* (minor and major pilin, *n*=2) (Fig. 8).

Most of the strains (*n*=67) harbour *comS*-I*–comR*-I genes and thus probably exhibit pherotype I. Pherotype II is found in 24 strains and pherotype III in only four strains (Fig. 8). One strain (strain 450) has a *comS* gene encoding a new variant of a pheromone (MNFIKAFTTFGTGWDWWWKG sequence) that we called *comS*-If. Interestingly, one genome contains two *comS* genes (one *comS*-Ic and one *comS*-III) and another one three *comR* genes (one comR-I, one comR-II and one comR-III). It would be interesting to test if these strains can produce the two peptides or sense three different pheromones respectively.

As described by Okura *et al*. [26], we found that the pherotype depends on the CCs (χ²=127.92, *P*=0.0005). Pherotype I is associated with CC1, CC16 and CC231 (100 % of the strains of these CCs have a pherotype I) and pherotype II with CC28 (91.7 % of the strains of this CC).

Variants (with <65 % amino acid identity with the ComFC protein of strain P1-7 of *S. suis*) were detected for the ComFC component of the DNA transport system in five strains (strains 450, 488, 561, 1452 and 1471) and for the ComYG pilin in 22 strains (corresponding to a large insertion in the middle of the protein which increases the length of the protein to 168 to 176 aa instead of 136 for strain P1-7 of *S. suis*).

## Conclusion

A large set of strains of *S. suis* isolated from humans, pigs and wild boars (*n*=102) in France was subjected to WGS. MLST data extracted from these genomes indicated that the most frequent CCs were CC1, CC28 and CC16. Through HCPC analysis, we identified a few virulence-associated factors that distinguish invasive CC1 strains from the other strains. This could be useful for epidemiology surveillance studies of this zoonotic pathogen. A plethora of AMR genes was found in the genomes, with several strains carrying up to seven AMR genes. Carriage strains were found to carry more AMR genes than invasive strains. The recently described *srpA* gene [62], which confers resistance to streptogramins A, lincosamides and to the pleuromutilin tiamulin, was found in 15 strains including two strains isolated from wild boars. Modifications in PBP 1a, PBP 2b, PBP 2x and MraY proteins were found in the penicillin-resistant isolates. A modification was also detected in the QRDR of GyrA and ParC in the fluoroquinolone-resistant isolate. Most of the identified AMR genes are carried by IMEs and new AMR gene–MGE associations were identified. In addition, the large majority of the strains have the full set of genes required for acquisition of extracellular DNA by natural transformation. These strains could thus take up AMR/virulence genes from other bacteria in the environment and serve as a reservoir of AMR genes for other bacteria also having this trait. This increases the risk of AMR dissemination. Reinforcing surveillance of AMR in *S. suis*, not only for invasive strains but also for carriage strains, would be useful since this bacterium is now recognized as a reservoir of AMR genes for other pathogens.

## supplementary material

10.1099/mgen.0.001224 Uncited Table S1.

10.1099/mgen.0.001224 Uncited Fig. S1.
