# Supplementary material for: Virulence genes, resistome and mobilome of Streptococcus suis strains isolated in France
Source: Microb Genom. 2024 Mar 28;10(3):001224. doi: 10.1099/mgen.0.001224 (PMC10995628; doi:10.1099/mgen.0.001224)
Supplement: Uncited Fig. S1. [file mgen-10-01224-s002.pdf]

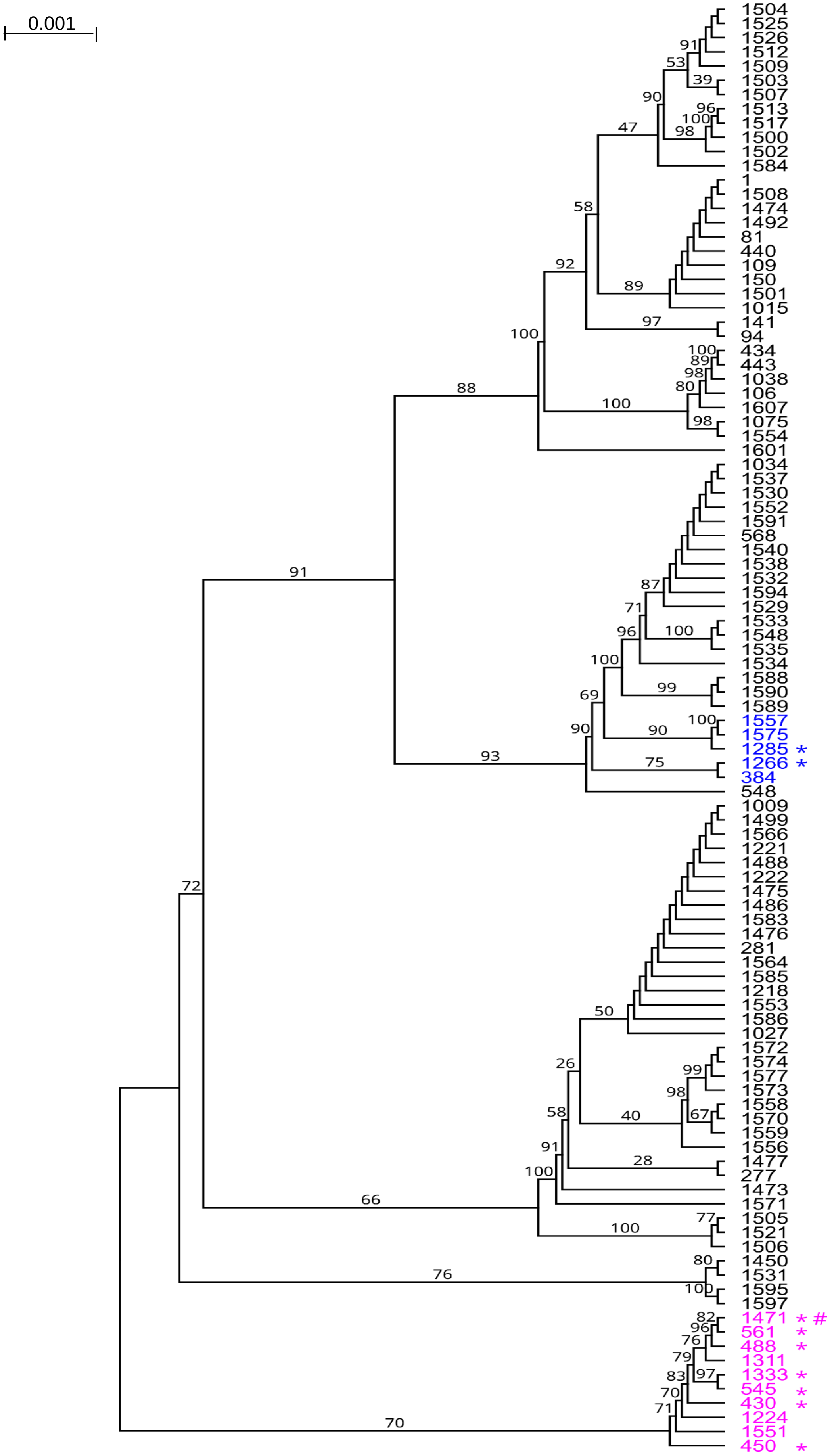

Supplementary figure S1: Genetic relatedness of the 102 genomes of French isolates of *Streptococcus suis*. Core genes were extracted and aligned using Roary and used for phylogenetic inference by maximum likelihood using IQ-TREE with the ModelFinderPlus option (see 'Methods'). The two lineages that include penicillin (indicated by a star) and fluoroquinolone resistant (indicated by a hash symbol) isolates are coloured in blue and pink in the tree.

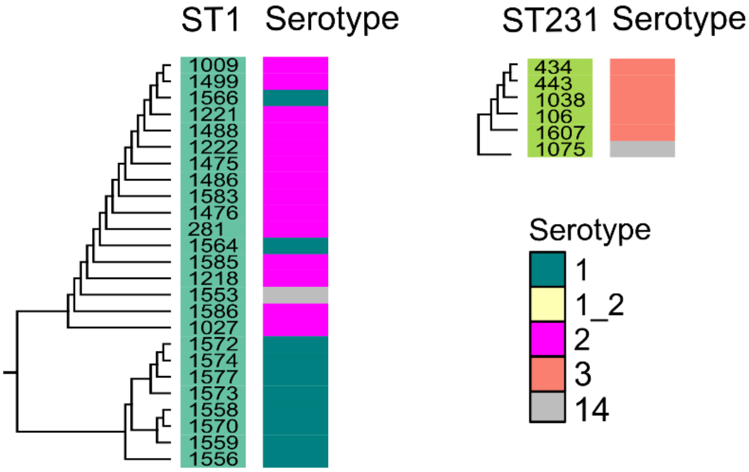

<u>Supplementary figure S2</u>: Examples of probable events of capsular switching among the *S. suis* strains studied.

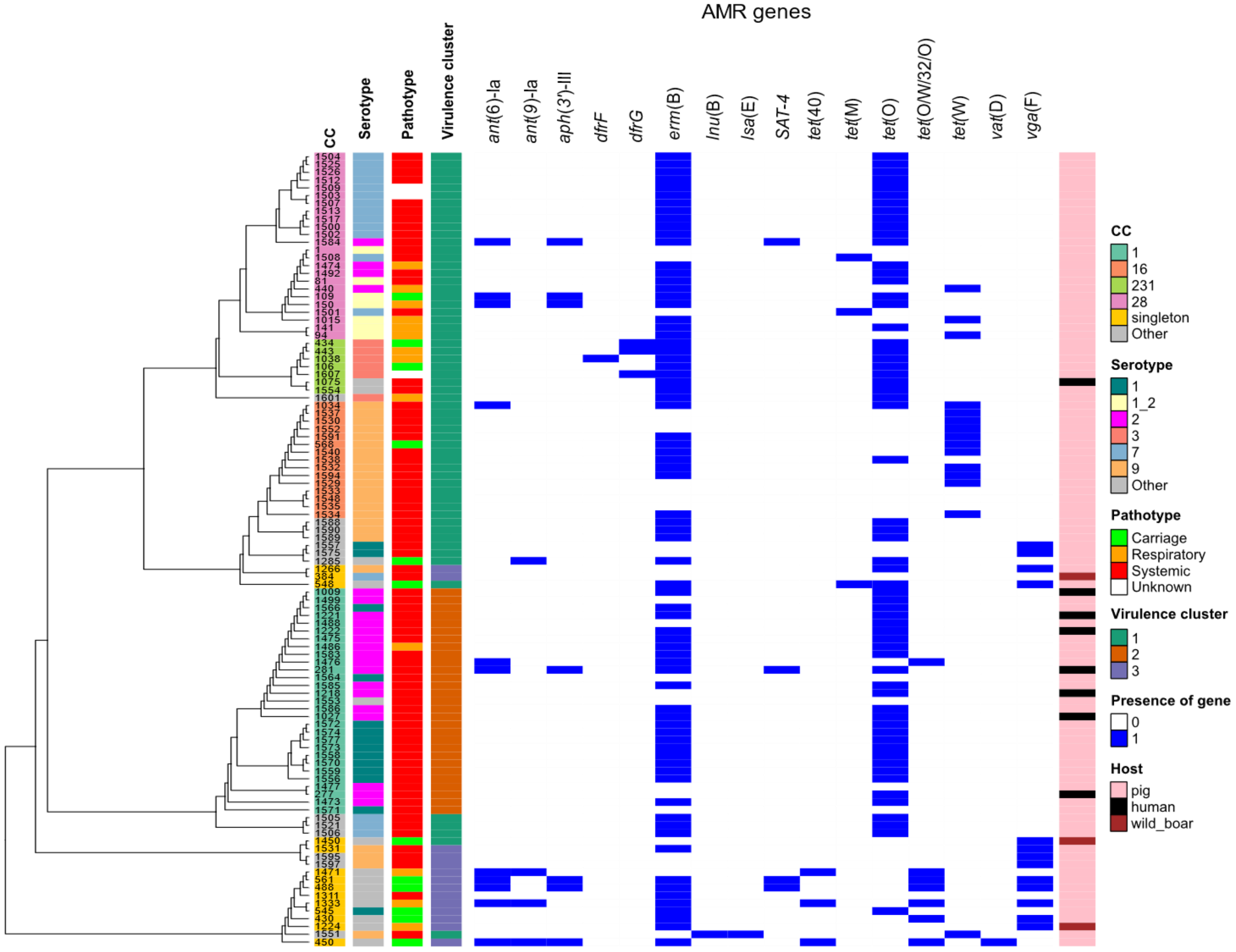

<u>Supplementary figure S3</u>: Heatmaps of (i) clonal complex (CC), (ii) serotype, (iii) pathotype, (iv) antimicrobial resistance genes and (iv) host for the 102 genomes of French isolates of *Streptococcus suis*. Genomes have been grouped according to their phylogenetic distance determined by alignment of their core genes (see 'Methods') (as indicated by a phylogenetic tree at the left). The legends for the heatmaps are indicated on the right of the figure. The names of the strains are indicated in the CC heatmap. Hierarchical clustering (HCPC) enabled the classification of the genomes in three clusters according to their pattern of putative virulence genes. The presence of AMR genes is indicated in blue.

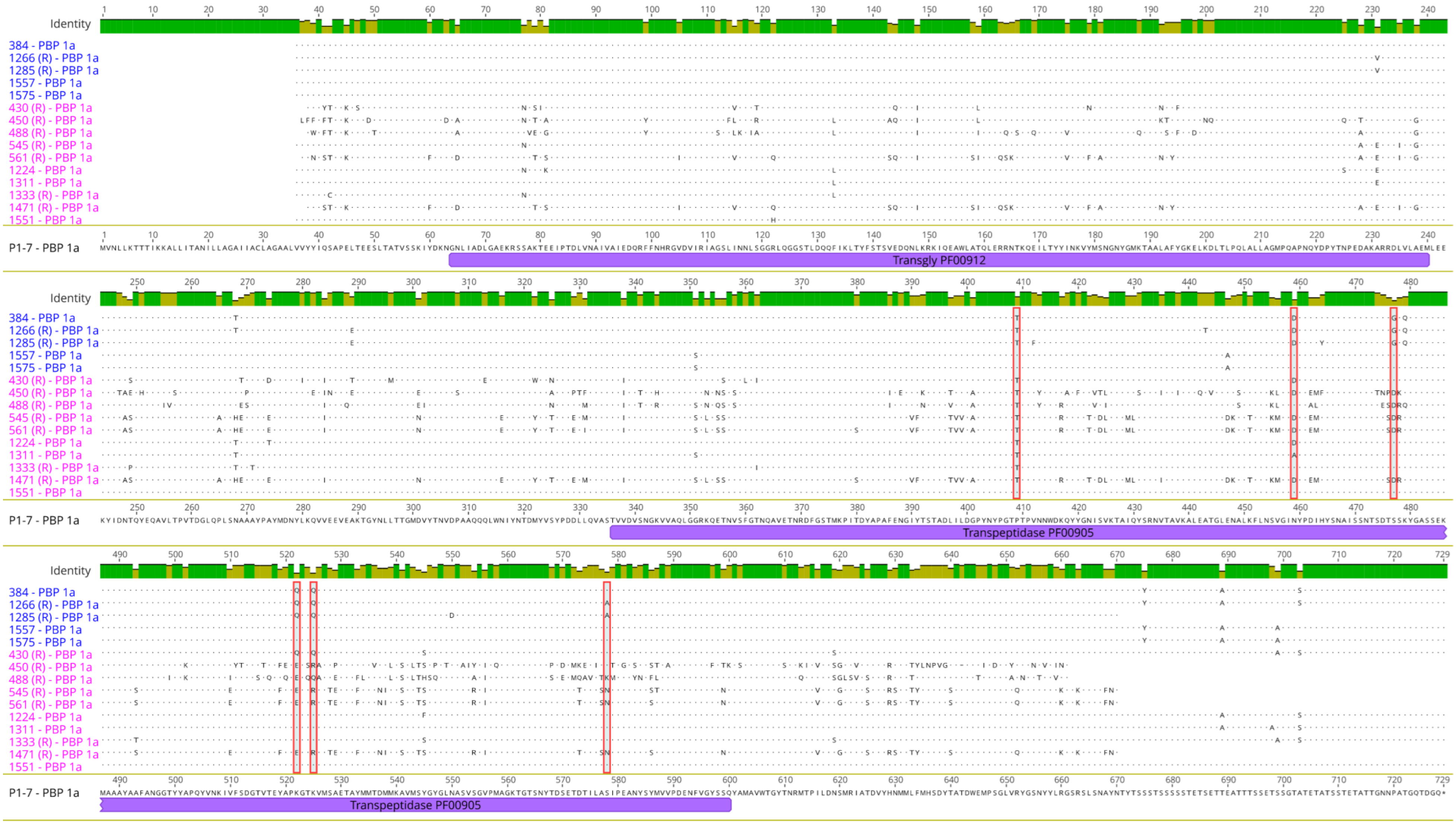

Supplementary figure S4: Analysis of the modifications in the PBP 1a protein in the nine penicillin resistant *S. suis* isolates (indicated by “R” after the name of the strain) described in this work. These isolates belong to different lineages (coloured in blue and pink). Alignments include PBP 1a proteins of penicillin susceptible strains close to the resistant isolates and the PBP 1a protein of *S. suis* P1-7 as reference (shown below the other sequences). Only discrepancies with the P1-7 reference sequence are shown. Modifications located in the Transpeptidase domain (shown in purple) that are present in at least one third of the resistant isolates and absent in at least half of the susceptible strains are indicated by a red box. The figure was generated using Geneious Prime 2023.2.1 (Biomatters).

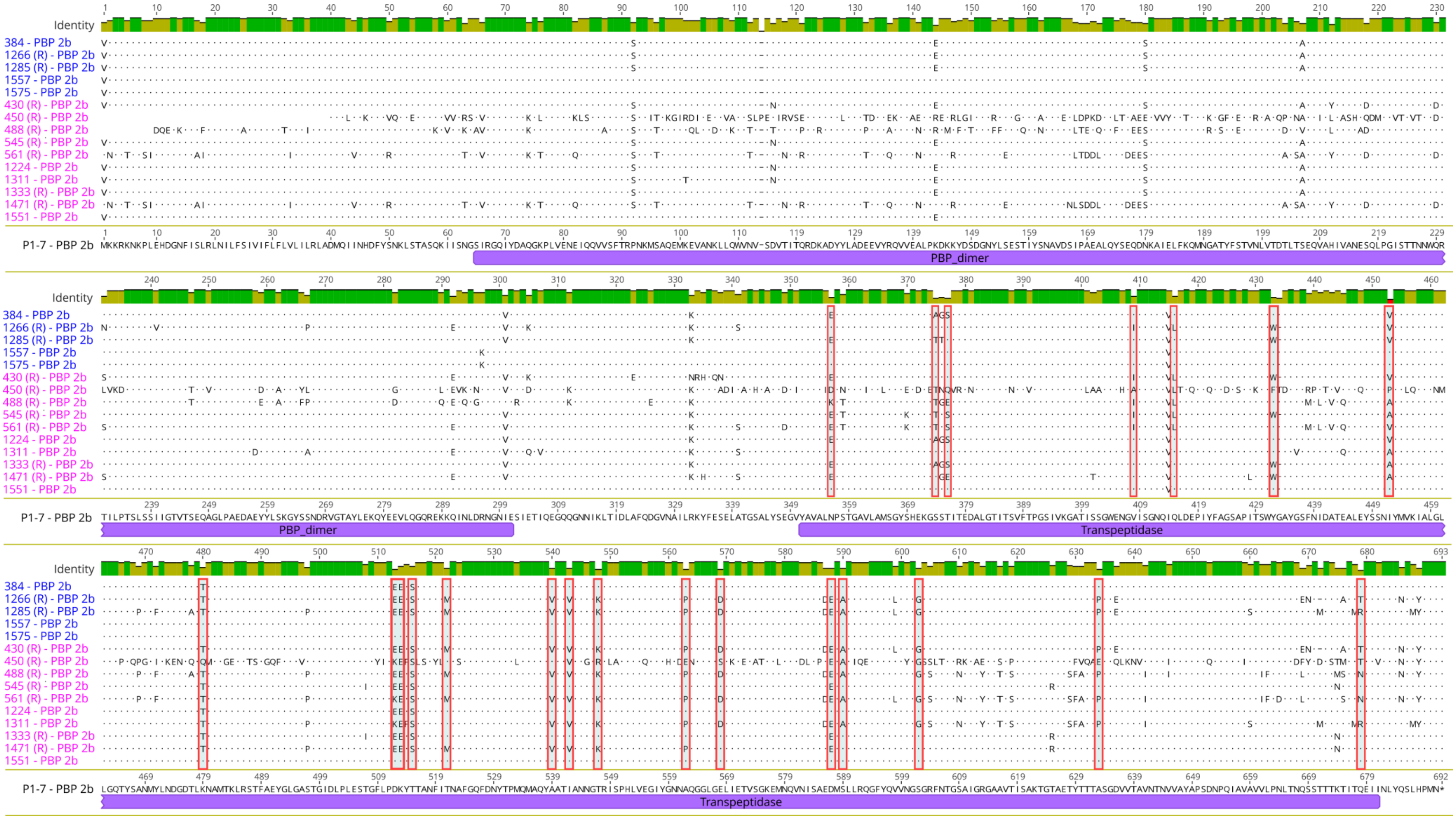

Supplementary figure S5: Analysis of the modifications in the PBP 2b protein in the nine penicillin resistant *S. suis* isolates (indicated by "R" after the name of the strain) described in this work. These isolates belong to different lineages (coloured in blue and pink). Alignments include PBP 2b proteins of penicillin susceptible strains close to the resistant isolates and the PBP 2b protein of *S. suis* P1-7 as reference (shown below the other sequences). Only discrepancies with the P1-7 reference sequence are shown. Modifications located in the Transpeptidase domain (shown in purple) that are present in at least one third of the resistant isolates and absent in at least half of the susceptible strains are indicated by a red box. The figure was generated using Geneious Prime 2023.2.1 (Biomatters).

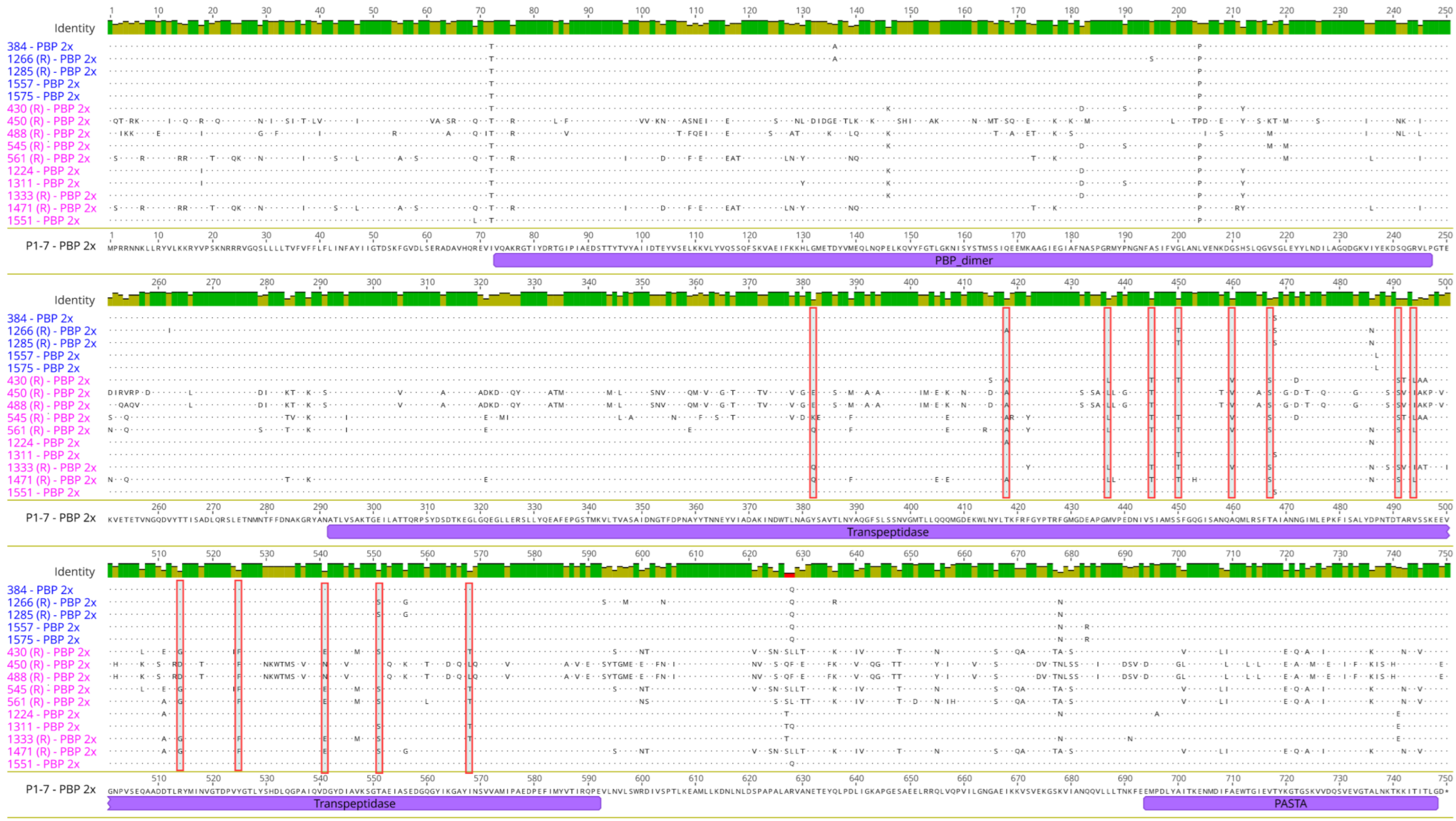

<u>Supplementary figure S6</u>: Analysis of the modifications in the PBP 2x protein in the nine penicillin resistant *S. suis* isolates (indicated by "R" after the name of the strain) described in this work. These isolates belong to different lineages (coloured in blue and pink). Alignments include PBP 2x proteins of penicillin susceptible strains close to the resistant isolates and the PBP 2x protein of *S. suis* P1-7 as reference (shown below the other sequences). Only discrepancies with the P1-7 reference sequence are shown. Modifications located in the Transpeptidase domain (shown in purple) that are present in at least one third of the resistant isolates and absent in at least half of the susceptible strains are indicated by a red box. The figure was generated using Geneious Prime 2023.2.1 (Biomatters).

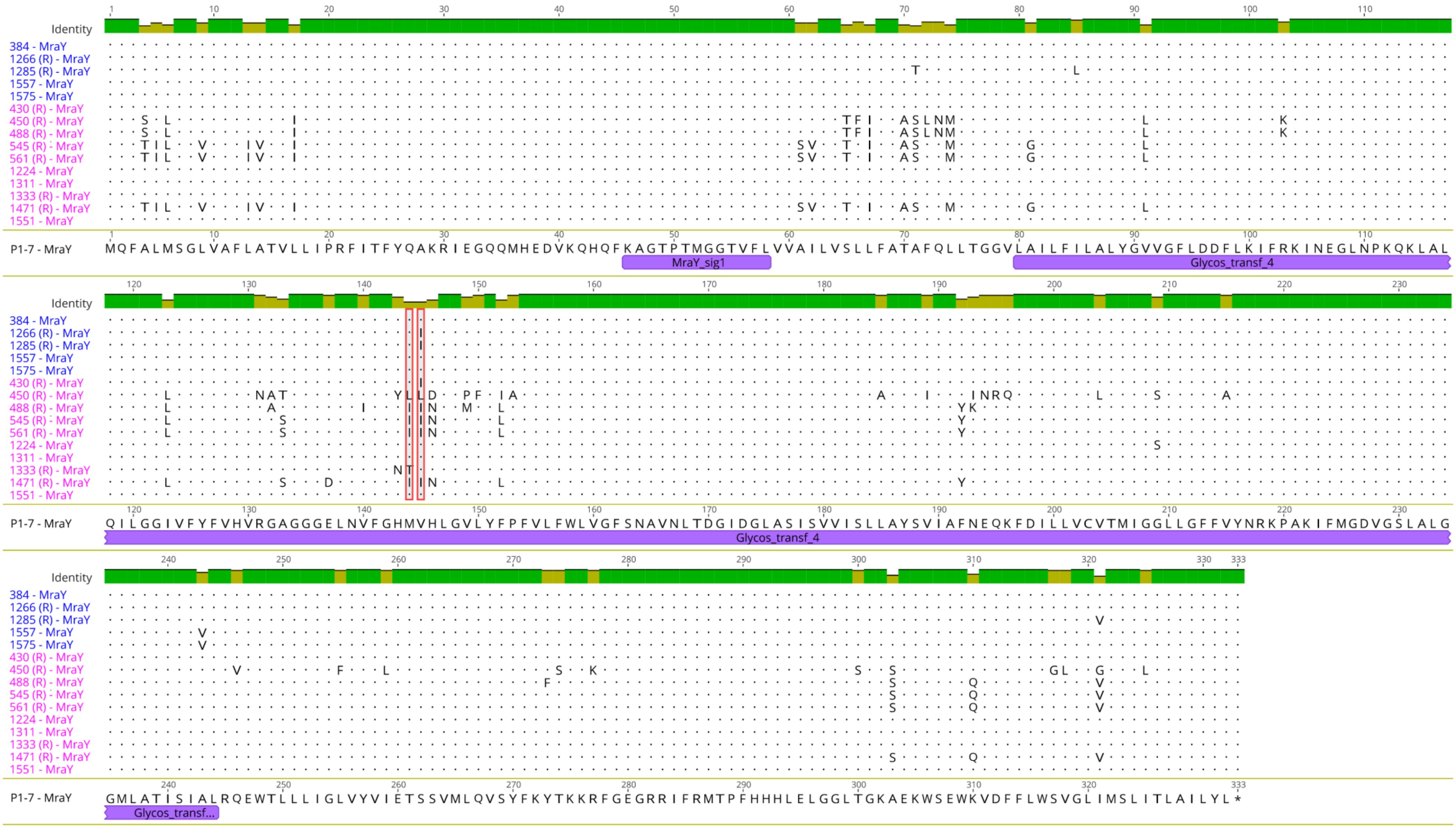

Supplementary figure S7: Analysis of the modifications in the MraY protein in the nine penicillin resistant *S. suis* isolates (indicated by "R" after the name of the strain) described in this work. These isolates belong to different lineages (coloured in blue and pink). Alignments include MraY proteins of penicillin susceptible close to the resistant isolates and the MraY protein of *S. suis* P1-7 as reference (shown below the other sequences). Only discrepancies with the P1-7 reference sequence are shown. Modifications located in the glycosyl transferase domain (shown in purple) that are found in at least one third of the resistant isolates and absent in at least half of the susceptible strains are indicated by a red box. The figure was generated using Geneious Prime 2023.2.1 (Biomatters).

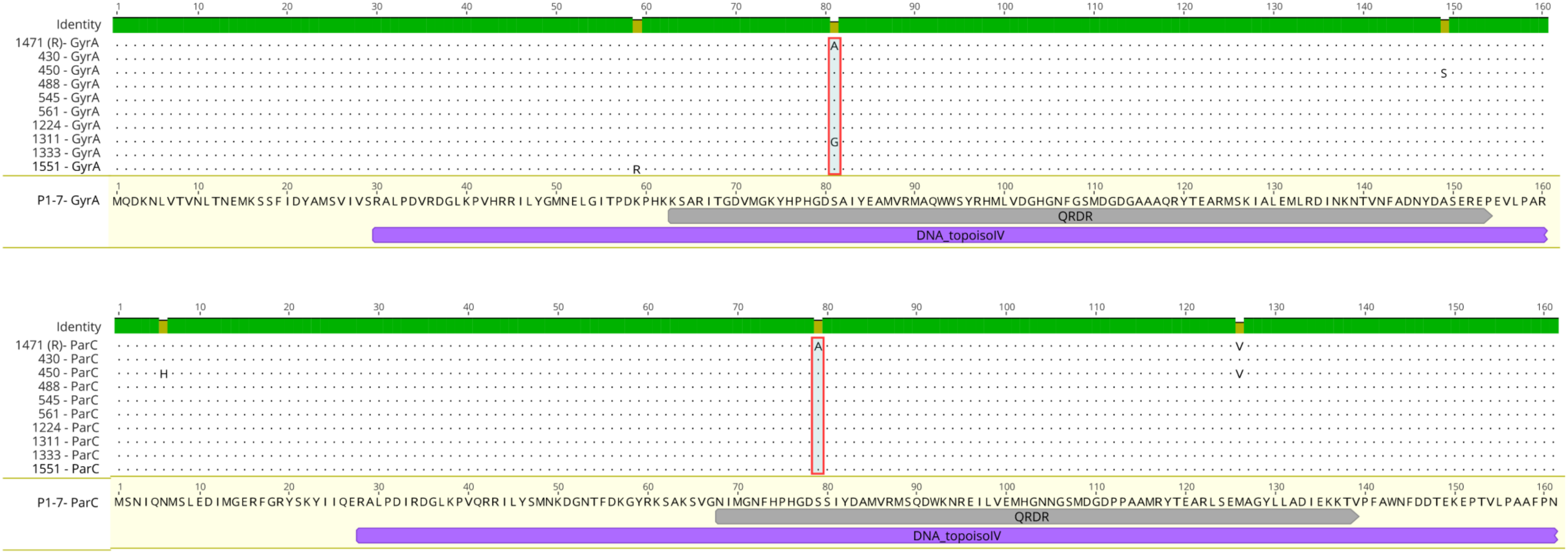

Supplementary figure S8: Analysis of the modifications in the Quinolone Resistance Determining Region (QRDR) of the sub-unit A of the gyrase (GyrA) and the sub-unit A of the topoisomerase IV (ParC) in the fluoroquinolone resistant *S. suis* isolate 1471 (indicated by "R" after the name of the strain) described in this work. Alignments include the QRDR of GyrA and ParC proteins of fluoroquinolone susceptible strains close to the resistant isolate and the GyrA and ParC QRDR of *S. suis* P1-7 as reference (shown below the other sequences). Only discrepancies with the P1-7 reference sequence are shown. Modifications located in the QRDR region (shown in grey) are indicated by a red box. The figure was generated using Geneious Prime 2023.2.1 (Biomatters).
